# Automatic small bowel tumor diagnosis by using multi-scale wavelet-based analysis in wireless capsule endoscopy images

**DOI:** 10.1186/1475-925X-11-3

**Published:** 2012-01-11

**Authors:** Daniel C Barbosa, Dalila B Roupar, Jaime C Ramos, Adriano C Tavares, Carlos S Lima

**Affiliations:** 1 Industrial Electronics Department, University of Minho, Portugal; 2 Gastroenterology Department, Hospital Santo Antonio dos Capuchos, Lisbon, Portugal

## Abstract

**Background:**

Wireless capsule endoscopy has been introduced as an innovative, non-invasive diagnostic technique for evaluation of the gastrointestinal tract, reaching places where conventional endoscopy is unable to. However, the output of this technique is an 8 hours video, whose analysis by the expert physician is very time consuming. Thus, a computer assisted diagnosis tool to help the physicians to evaluate CE exams faster and more accurately is an important technical challenge and an excellent economical opportunity.

**Method:**

The set of features proposed in this paper to code textural information is based on statistical modeling of second order textural measures extracted from co-occurrence matrices. To cope with both joint and marginal non-Gaussianity of second order textural measures, higher order moments are used. These statistical moments are taken from the two-dimensional color-scale feature space, where two different scales are considered. Second and higher order moments of textural measures are computed from the co-occurrence matrices computed from images synthesized by the inverse wavelet transform of the wavelet transform containing only the selected scales for the three color channels. The dimensionality of the data is reduced by using Principal Component Analysis.

**Results:**

The proposed textural features are then used as the input of a classifier based on artificial neural networks. Classification performances of 93.1% specificity and 93.9% sensitivity are achieved on real data. These promising results open the path towards a deeper study regarding the applicability of this algorithm in computer aided diagnosis systems to assist physicians in their clinical practice.

## Background

### Capsule Endoscopy

#### General Considerations

The innovation of wireless capsule endoscopy (CE) has revolutionized the investigation and management of patients with suspected small bowel disease [1]. Since its introduction, in the year 2000, a new chapter in the small bowel examination was opened, as this new technology allows the visualization of the entire gastrointestinal (GI) tract, reaching places where conventional endoscopy is unable to. In fact, conventional endoscopy presents some important limitations in the diagnosis of small bowel problems, since it is limited to the upper GI tract, at the duodenum, and to lower GI tract, at terminal ileum. Therefore, prior to the wireless capsule endoscopy era, the small intestine was the conventional endoscopy's last frontier, because it could not be internally visualized directly or in it's entirely by any method [2]. The small intestine accounts for 75% of the total length and 90% of the surface area of the gastrointestinal tract [3]. In adults it measures about 570 cm at *post mortem*, which is substantially longer than conventional video endoscopes (100-180 cm) [3]. Push enteroscopy (PE) is an effective diagnostic and therapeutic procedure, although it only allows exploration of the proximal small bowel [4]. Intraoperative enteroscopy is the most complete but also the most invasive means of examining the small bowel [5]. On the other hand, CE is a simple, non-invasive procedure that is well accepted by the patient and can be performed on an outpatient basis, allowing simultaneously the visualization of the entire GI tract. This technique is especially successful in finding bleeding regions, Crohn's disease and suspected tumors of the small bowel [2,6].

The first commercially-available wireless video capsule was the M2A^TM ^(by Given Imaging Ltd., Yoqneam, Israel), a pill-like device (11 mm × 26 mm), which contains a miniaturized camera, a light source and a wireless circuit for the acquisition and transmission of signals [7]. The capsule is passively propelled trough the entire GI tract, through peristalsis, capturing images at a rate of two frames per second. Image features include a 140° field of view, 1:8 magnification allowing visualization of individual villi, 1-30 mm depth of view and a minimum size of detection of about 0.1 mm.

#### Examination Procedure

While conventional endoscopy diagnosis procedure consists in an exam that uses a flexible endoscope, with a video camera in the distal tip, to acquire intra-corporeal images from the GI tract as the endoscope is pushed into the patient's body, a capsule endoscopy exam relies in a small pill-like device, which is ingested and propelled by natural peristalsis through the GI tract, acquiring images while it travels [7]. Therefore, major limitations of the conventional endoscopy are solved, since great skill and concentration are required to navigate a conventional endoscope. Furthermore, and since no drugs are administered, some investigators maintain that the use of the capsule camera is a more physiological form of endoscopy than conventional push enteroscopy [8]. By the time battery power expires, after about 8 transit hours through the GI tract, the camera will have captured about 55,000 images, which are transmitted to the hard drive in a belt worn by the patient [8]. The capsule is excreted in the patient's stool, usually within 24-48 h, and not reused [9]. The time required to a physician to analyze the resulting video is, on average, 40-60 min [9]. The reading time and interpretation of CE exams is very time consuming given that more than 50,000 images have to be reviewed [10,11], which contributes to the high cost of a CE exam [12]. Thus, a computer assisted diagnosis tool to help the physicians to evaluate CE exams faster and more accurately is an important technical challenge and an excellent economical opportunity.

### Small Bowel Tumor Diagnosis using Capsule Endoscopy

After the introduction of CE, it was discovered that prevalence and malignancy rates for small bowel tumors are much higher than previously reported and that the early use of CE can lead to earlier diagnoses and reduced costs, contributing hopefully to cancer prevention [2]. A small bowel tumor is diagnosed in approximately 2.5-9% of patients submitted to CE, indicating that the frequency of these neoplasms is considerably higher than previously thought. At least 50% of small intestine tumors identified with CE are malignant [9]. However, the early diagnosis of small bowel tumor is difficult, because signs are vague and laboratory tests are unhelpful [13]. However, obscure GI bleed can be an earlier symptom and a key factor for an early diagnosis of these lesions [2]. Small bowel tumors are a significant finding at CE and are often missed by other methods of investigation. Thus, the automatization of the analysis CE can greatly help in a more efficient screening for these tumor lesions.

### Previous Work

Current approaches rely in the fact that alterations in the texture of the small bowel mucosa can be used in automatic detection methods of abnormalities, which potentially indicate disease. These alterations are just what the physicians usually search for. For instance, Maroulis *et al. *and Karkanis *et al. *proposed two different methods based on the analysis of textural descriptors of wavelet coefficients in colonoscopy videos [14,15]. Indeed texture extraction algorithms can be used as feature sources of classifiers, in order to develop automatic classification schemes for CE video frames evaluation. Kodogiannis *et al. *proposed two different schemes to extract features from texture spectra in the chromatic and achromatic domains [16]. Although presented for a slightly different event detection, the works of Cunha *et al. *and Mackiewicz *et al. *suggest that a significant reduction of the viewing time can be achieved by automatic topographic segmentation the capsule endoscopic videos [17,18]. Szczypinski *et al. *have recently proposed a different and very interesting concept to aid clinicians in the interpretation of capsule endoscopic videos [19]. They propose the use of a model of deformable rings to compute motion-descriptive characteristics and to produce a two-dimensional representation of the GI tract's internal surface. From these maps, certain characteristics that indicate areas of bleeding, ulceration and obscuring froth can be easily recognized, allowing therefore the quick identification of such abnormal areas. Recently, a different approach has also been proposed by Iakovidis *et al. *to reduce the capsule endoscopic reading times, through the use of an unsupervised image mining technique [20]. Using a different rationale than the typical viewing time reduction, Karargyris and Bourbakis have recently proposed a method to enhance the video and therefore improve the viewing of the digestive tract, leading to a richer, more qualitative and efficient CE examination [21]. The detection of abnormalities, with special incidence in blood presence, in CE frames through computational approaches has been indeed a particularly active topic in the last few years [22-25]. For further notes on the available methodologies for CE image processing, the reader is advised to consult the recent review by Karargyris and Bourbakis [26]. In authors' previous work [27-30], different methods are proposed for classification of capsule endoscopic video frames based on statistical measures taken from texture descriptors of co-occurrence matrices, using the discrete wavelet transform to select the bands with the most significant texture information for classification purposes. Furthermore, the measurement of the non-Gaussianity of these statistical texture descriptors regarding marginal distributions was used in [29], in a classification scheme to identify abnormal frames. This paper proposes extending this approach to the joint distribution modeling, allowing to further explore the texture patterns in CE frames, having however the drawback of strongly increasing the dimensionality of the observation vector.

### Proposed Approach

**Figure 1:**
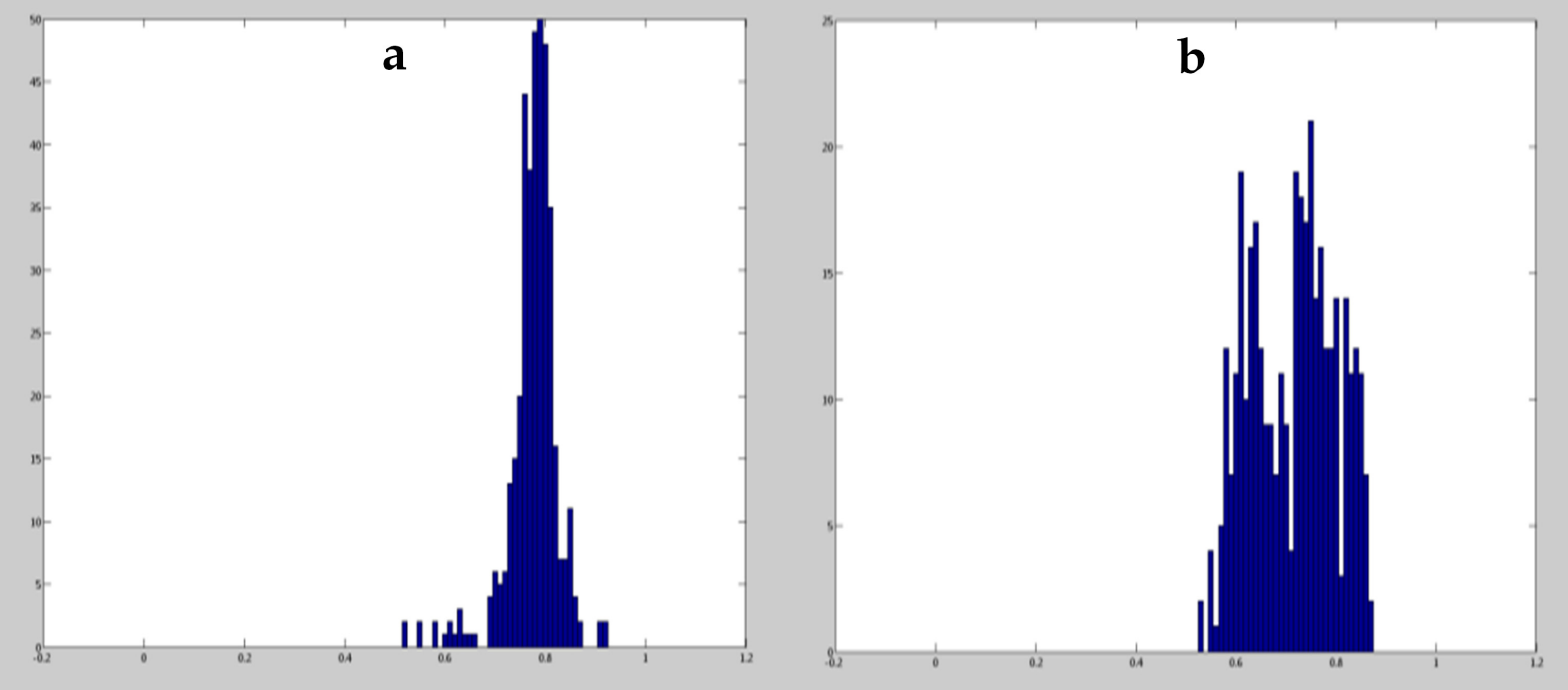
**Non-Gaussianity of texture descriptors**. Distribution of F1 texture descriptors for a set of 300 frames (a: normal capsule endoscopic frames, b: abnormal (small bowel tumor) capsule endoscopic frames).

The algorithm proposed in this paper is based on combined information from both color and texture fields of the image and is an improvement of the approach followed in [28] and [29], taking into consideration the findings in [14] and [15]. In [14] and [15], it is observed that both the set of wavelet sub-bands {1, 2, 3} and {4, 5, 6} have significant texture information and perhaps both must be considered. Therefore texture description can perhaps be improved by using information from these two levels of focus. Assuming joint Gaussianity of each descriptor observed at different colors and different scales, information from both levels of focus can be inserted in the statistical model by correlating the observations. Texture characterization by statistical modeling of texture descriptors at different scales and colors is one of the novelties of this paper. However by inspecting marginal distributions of texture descriptors we can find several examples of non-Gaussianity specially in tumoral frames as can be seen in Figure 1. This fact claims for Higher Order Statistics (HOS), since it is well known from statistics that marginal non-Gaussianity always origins joint non-Gaussianity. HOS applied in the context of texture characterization by using the multi-scale concept in synthesized images was proposed in [28] but only applied in the marginal distributions. The extension of this concept to the joint distributions is another novelty of this paper, which increases significantly the dimensionality of the observation vectors.

**Figure 2:**
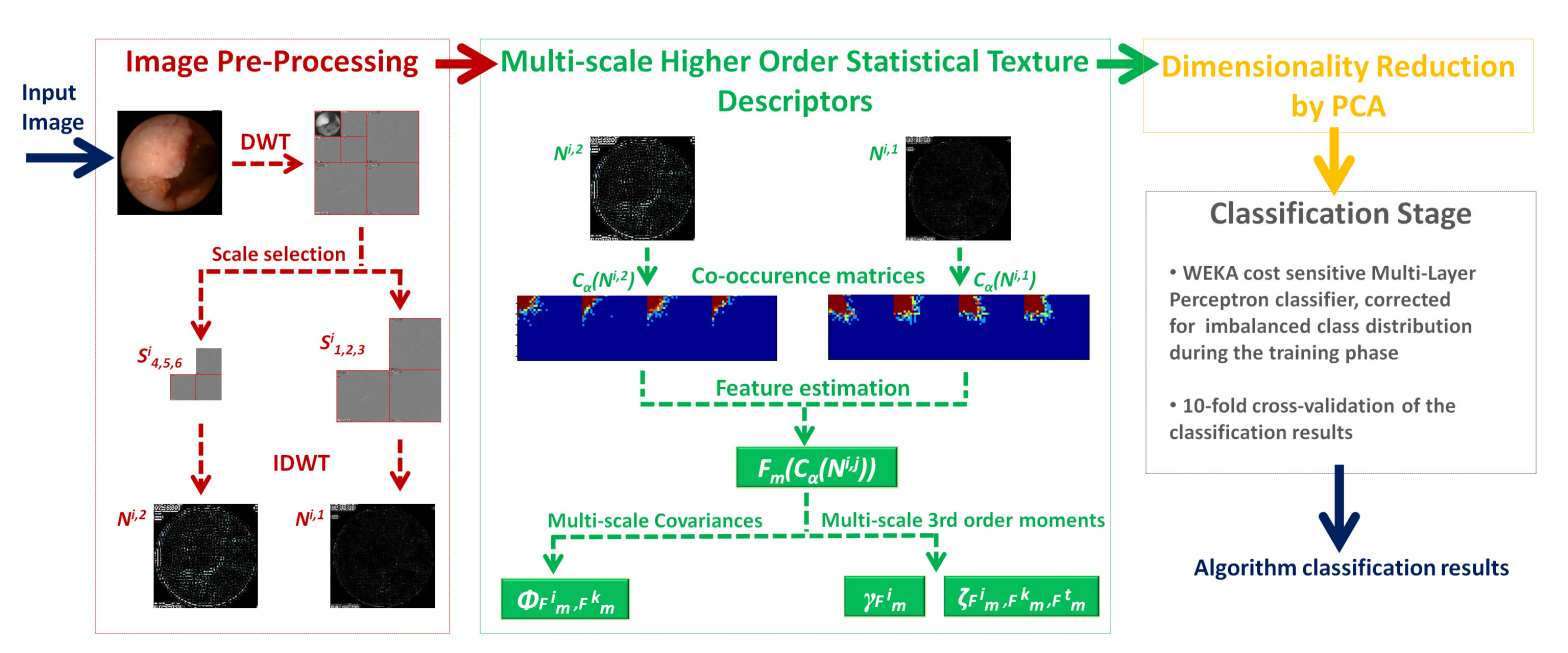
**Algorithm Flowchart**. Data flow throughout the algorithm key blocks. An initial pre-processing step is applied to the image in order to synthesize two images containing only the texture details corresponding to the medium and high frequency content of the original image. In order to compute the proposed texture descriptors, co-occurrence matrices are computed for each synthesized image and several features are extracted from these matrices. Then, multi-scale higher order statistical modeling is applied to extract the proposed texture descriptors. An optional dimensionality reduction of the feature vector can be applied prior to the classification stage.

The method proposed in this paper is then based on the multi-scale higher order statistical features of two images reconstructed from the wavelet coefficients of the selected wavelet bands, which contain the most important texture information for classification purposes. Furthermore, and to reduce the dimensionality of the feature set, PCA is applied, being this the third novel point of the paper justified by the high dimensionality of the feature vector when compared with the amount of training data. These features are the input of a Multi-Layer Perceptron classifier, in a classification scheme used to classify real data gathered at the Hospital dos Capuchos. A flowchart with the key processing blocks of the proposed algorithm is illustrated in Figure 2.

## Methods

### General considerations

Although texture can be visually classified as fine, course, grained, smooth, etc., its mathematical definition is not trivial. Nonetheless, it is clear that the textured information in a image corresponds to its medium/high frequency content. Therefore, the texture can be seen as a multi-scale phenomena and thus appropriate computational tools are required to deal with its quantification and analysis. The Discrete Wavelet Transform (DWT) is a mathematical tool that allows a spatial/frequency representation by decomposing the image in different scales with different frequency content. Thus, the DWT is a multi-resolution representation of the information within the image, being therefore well suited to deal with multi-scale phenoma as the texture content of an image. In the present work, we use the DWT to select the appropriate frequency content to further proceed to the analysis of the texture content of a given image. This information is mostly present in the scales corresponding to medium/high frequency content (sub-bands {4, 5, 6} and sub-bands {1, 2, 3}, respectively), and thus an image is synthesized from each of these scales. Each synthesized image will thus possess relevant texture information at the selected scale of detail. Nevertheless, one should recall that the sub-band selection is dependent on the image resolution, which imply that a pre-regularization step may be added in order to account for frames with different image resolution.

Once the relevant information is selected by using DWT, several statistical texture descriptors can be extracted. Since texture is a phenomena involving spatial patterns among neighbor pixels, it can be properly exploited using co-occurrence matrices. These are usually built by estimating the second order joint-conditional probability density function *f*(*i*, *j*, *d*, *θ*), which is computed by counting all pairs of pixels at distance *d*, having pixel intensity of color levels *i *and *j *at a given direction *θ*. The angular displacement used is the set {0, *π*/4,*π*/2, 3 *π*/4}. Several features can be extracted from co-occurrence matrices, corresponding to statistical descriptors containing second order color level information, which are mostly related to the human perception and discrimination of textures [31]. In the present work only 4 statistical measures are considered among the 14 originally proposed [31]. They are angular second moment (*F*_1_), correlation (*F*_2_), inverse difference moment (*F*_3_), and entropy (*F*_4_), representing the homogeneity directional linearity, smoothness and randomness of the co-occurrence matrix, defined respectively as:

(1) $$F_{1}=\overset{N_{g}}{\underset{i=1}{\sum }}\overset{N_{g}}{\underset{j=1}{\sum }}p{\left(i,j\right)}^{2},$$

(2) $$F_{2}=\frac{\sum_{i=1}^{N_{g}}\sum_{j=1}^{N_{g}}\left(i\cdot j\right)\cdot p\left(i,j\right)-\mu_{x}\cdot \mu_{y}}{\sigma_{x}\cdot \sigma_{y}},$$

(3) $$F_{3}=\overset{N_{g}}{\underset{i=1}{\sum }}\overset{N_{g}}{\underset{j=1}{\sum }}\frac{1}{1+\left(i-j\right)}p\left(i,j\right)\;\;\;,j\neq i+1,$$

(4) $$F_{3}=\overset{N_{g}}{\underset{i=1}{\sum }}\overset{N_{g}}{\underset{j=1}{\sum }}p\left(i,j\right)log_{2}p\left(i,j\right)\;\;\;,p\left(i,j\right)\neq 0,$$

where *p*(*i*,*j*) is the *ij*^*th *^entry of the normalized co-occurrence matrix, *N*_*g *_the number of gray levels of the synthesized image and *μ*_*x*_, *μ*_*y*_, σ_*x *_and *σ*_*y *_are the means and standard deviations of the marginal probabilities *p*_*x*_(*i*)/*Py*(*j*) obtained by summing up the rows/columns of the matrix *p*(*i,j*). In the ambit of this paper these features were obtained from pre-processed images, which are synthesized from the inverse wavelet transform of the wavelet transform of source images where information not relevant for texture analysis was discarded in the wavelet domain, as described in the sequel. HOS in the color-scale space was added to cope with joint non-Gaussianity that certainly happens under marginal non-Gaussianity cases. The resulting feature vector will allow to capture enough information in order to identify characteristic texture patterns from normal and abnormal small bowel tissue.

### Feature Extraction Algorithm

#### Image Pre-processing

The image pre-processing stage synthesizes two images containing only the most relevant textural information from the source image. The most relevant texture information often appears in the middle frequency channels [32]. Texture is the discriminating information that differentiates normal from abnormal lesions, regarding colorectal diagnosis [14,15,33,34], hence it might be extrapolated to small bowel diagnosis with similar characteristics.

The wavelet transform allows a spatial/frequency representation by decomposing the image in the corresponding scales. When the composition level decreases in the spatial domain it increases in the frequency domain providing zooming capabilities and local characterization of the image [35]. This spatial/frequency representation, which preserves both global and local information, seems to be adequate for texture characterization.

Color transformations of the original image *I *result in three decomposed color channels:

(5) $$I^{i},\;\;\;\;\;i=1,2,3,$$

where *i *stands for the color channel.

**Figure 3:**
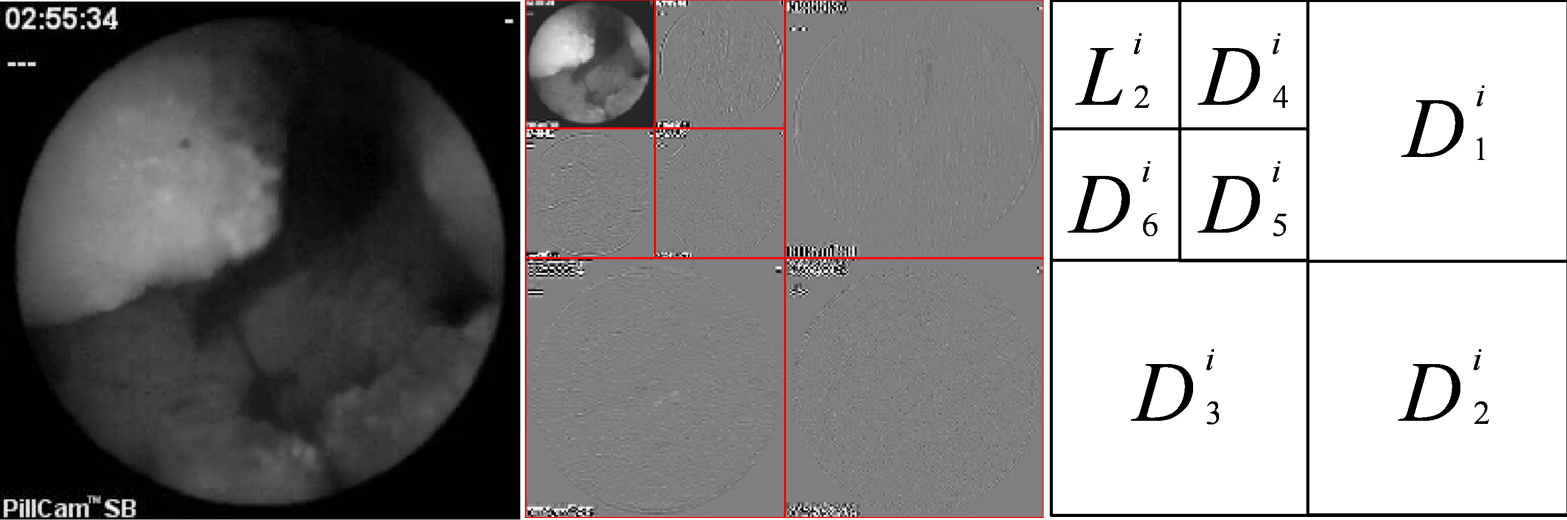
**2D DWT of a CE frame**. Example of two level discrete wavelet decomposition scheme of the original image for color channel *i*.

A two level discrete wavelet frame transformation is applied to each color channel, *I*^i^, as shown in Figure 3. This transformation results in a new representation of the original image by a low resolution image and the detail images. Therefore the new representation is defined as:

(6) $$W^{i}=\left\{L_{n}^{i},\;D_{l}^{i}\right\},\;\;\;\;\;n=2;\;\;l=1,2,3,4,5,6,$$

where *l *stands for the wavelet band and *n *is the decomposition level.

Since the textural information is better presented in the middle wavelet detailed channels, then second level detailed coefficients would be considered. However, the relatively low image dimensions (256 × 256) limit the representation of the details, becoming the first level more adequate, than previously expected, for texture representation [27]. Nevertheless an image resolution normalization might be required for different image resolutions. The image representation consists of the detail images produced from (6) for the values 1 = 1, 2, 3, 4, 5 and 6, as shown in Figure 3. This results in a set of 18 sub-images:

(7) $$D_{l}^{i},\;\;\;\;i=1,2,3;\;\;l=1,2,3,4,5,6.$$

For the extraction of the second order statistical textural information, co-occurrence matrices would be calculated over the eighteen different sub-images. However, and in order to diminish the dimension of the observation vectors, the image to be processed can be synthesized from inverse discrete wavelet transform (IDWT), with the coefficients of the large scales (lower frequencies) discarded. As the goal is to correlate texture descriptors obtained at different scales and colors, one image must be synthesized from each scale and color channel. The number of scales considered in this paper is 2. Therefore one image must be synthesized from bands {1, 2, 3} and a second image is synthesized from bands {4, 5, 6}, for each color channel. This procedure reduces the dimensionality of the observation vector by a factor of 3, since only six images need to be processed instead of the eighteen obtained in the wavelet domain. Previous results confirmed that the most relevant texture information is maintained with this approach [27,29]. It is important to emphasize that this selection is mainly related with the detail level selection. Indeed, the orientation information discarded in the IDWT synthesis process is not a major concern at this point. Furthermore the orientation information will be considered at the time of the co-occurrence matrices calculation. Let S be a matrix that has the selected wavelet coefficients at the corresponding positions and zeros in all other positions:

(8) $$S_{l}^{i}=D_{l}^{i},\;\;\;\;i=1,2,3;\;\;l=1,2,3\;\;\vee l=4,5,6.$$

Two new images, containing the most important texture information, are then synthesized from the selected wavelet bands, trough the inverse wavelet transform. Each image represents the original image at a different level of focus. Let *N*^*i,j *^where *j = *{1, 2} represents the level of focus, be the reconstructed images for each color channel:

(9) $$N^{i,1}=IDWT\left(S_{l}^{i}\right),\;\;\;\;i=1,2,3;\;\;\;l=1,2,3,$$

(10) $$N^{i,2}=IDWT\left(S_{l}^{i}\right),\;\;\;\;i=1,2,3;\;\;\;l=4,5,6,$$

where *i *stands for color channel, *l *for wavelet band and *IDTW*(·) is the inverse wavelet transform. Unfortunately the contribution of the detail information regarding texture characterization can not be visually confirmed since the lack of the coarse scale (low frequency content) retires almost all the visual information. In this regard we perhaps should rely in the well established knowledge that textural information is most present in the higher frequencies (image detail).

#### Multi-scale textural descriptors

For all the synthesized images *N*^*i,j *^co-occurrence matrices are calculated. These matrices capture spatial interrelations among the intensities within the synthesized image level. The co-occurrence matrices are estimated in four different directions resulting in 24 matrices:

(11) $$C_{\alpha }\left(N^{i,j}\right),\;\;\;\;\alpha =0,\pi /4,\pi /2,3\pi /4,$$

where *α *stands for the direction in the co-occurrence computation. Four statistical measures given by equations (1), (2), (3) and (4) are estimated for each matrix resulting in 96 texture descriptors:

(12) $$F_{m}\left(C_{\alpha }\left(N^{i,j}\right)\right),\;\;\;\;m=1,2,3,4,$$

where *m *stands for statistical measure.

#### Multi-scale Higher Order Statistical Modeling of Texture Features

Since each feature extracted from the co-occurrence matrices represents a different property of the synthesized image, it is expectable that similar textures have close statistical distributions and consequently they present similar features. This similarity between features can be statistically modeled in a two-dimensional space (color-scale space) since features can be simultaneously observed in the three channel colors for both levels of focus.

While the texture descriptors can be considered statistically independent [31], their occurrence together in the three color channels for both levels of focus is likely to be correlated. The correlation between two descriptors measures their tendency to vary together and constitutes the sufficient statistics when the multivariate density is normally distributed.

Therefore we propose to model the texture descriptors as a multivariate Gaussian distribution in the color-scale space. In this framework 21 correlations must be computed for each texture descriptor as shown in Table 1. There, capital letters stand for the high frequency image while lowercase letters stand for the medium frequency image. Prom Table 1 and considering 4 texture descriptors, 84 correlations must be computed for each endoscopic capsule image.

**Table 1:** Correlations computed for Multi-Scale analysis

	H	S	V	h	s	v
H	#	#	#	#	#	#

S		#	#	#	#	#

V			#	#	#	#

h				#	#	#

S					#	#

**V**						#

This table highlights how the multi-scale correlation schemes are performed.

Note that H stands for the texture descriptors extracted from the high-frequency content of the Hue channel, while s stands for the texture descriptors extracted from the medium-frequency content of the Saturation channel.

Second order statistics is a well established theory that is completely adequate to represent random vectors. Nevertheless, it is limited by the assumptions of Gaussianity, linearity, stationarity, etc. One of the main properties of the multivariate normal distributions is that the marginal distributions are also normal although the converse is not necessarily true. However, it is very common in practical applications to assume Gaussianity in order to obtain mathematical tractability or to alleviate computational load. Many multivariate statistics used in practice converge in distribution to a multivariate normal, which is acceptable regarding the multivariate central limit theorem. For the most of the Engineering applications, in spite of sometimes the distributions tend to be clearly non-Gaussian, modeling non-Gaussianity usually improves the performance only marginally. This is partially due to the fact that usually higher order moments need much more training data to be accurately modeled than second order moments. For the current application, statistical descriptors tend clearly to be non-Gaussian, especially for pathological cases as shown in Figure 1[30].

HOS characterized by higher order moments are adequate to model non-Gaussian distributions under the assumption that all the moments are finite and so their knowledge is in practice equivalent to the knowledge of their probability function [36]. Third and fourth order moments have precisely meaning in separating Gaussian from other distributions. The third central moment:

(13) $$\mu_{3}=E\left\{{\left(x-\bar{x}\right)}^{3}\right\}$$

gives a measure of assymmetricity of the probability density function around their mean (skewness), while the fourth central moment gives a measure of the peaky structure of the distribution when compared with the Gaussian. Higher than fourth order moments are used seldom in practice, hence not tried in the ambit of this paper. However, and given the authors previous experience [29] and preliminary tests with the proposed algorithm, only the second and third order moments were described in the present work, given that the fourth order moment does not lead to a relevant increase in the classification performance. The second order moments or correlation of the same statistical measure between different color channels and using both scales is computed as:

(14) $$\phi_{F_{m}^{i},F_{m}^{k}}=\sum_{a}F_{m}\left(C_{\alpha }\left(N^{i,j}\right)\right)\cdot F_{m}\left(C_{\alpha }\left(N^{k,j}\right)\right),$$

where *i *and *k *refers to the different color channels and *j *refers to selected wavelet band, and can therefore take the values of 1 and/or 2. This approach results in the computation of 84 coefficients, 21 different correlations computed for each descriptor as shown in Table 1. The third order moments are computed as:

(15) $$\gamma_{F_{m}^{i}}=\underset{a}{\sum }{\left(F_{m}\left(C_{\alpha }\left(N^{i,j}\right)\right)\right)}^{3},$$

(16) $$\begin{array}{c} \zeta_{F_{m}^{i},F_{m}^{k},F_{m}^{t}}=\sum_{a}F_{m}\left(C_{\alpha }\left(N^{i,j}\right)\right)\cdot F_{m}\left(C_{\alpha }\left(N^{k,j}\right)\right)\\ \;\;\;\;\;\;\;\;\;\;\;\;\;\;\;\;\;\;\;\;\;\;\;.F_{m}\left(C_{\alpha }\left(N^{t,j}\right)\right),\;\;\;i\neq k\neq t. \end{array}$$

From (15) six third order moments are computed for each descriptor, since six color matrices are available, three in each wavelet scale. Equation (16) provides a larger number of possible combinations, all of them required for modeling the joint probability density function of each descriptor when simultaneously observed in the three colors at each scale. However, and since that correlation of the textural information in the same color channel at different detail levels is already implicitly calculated in the second order moments, the third order moments corresponding to combinations that arise from the same color channel at different scales (eg. HhV) were not calculated in order to diminish the observation vector. Preliminary tests have shown that this approximation does not decrease the classification performance. Therefore the third order moments were calculated for the following cases: HSV, hSV, HsV, HSv, hsv, Hsv, hSv and hsV, accordingly with the nomenclature of Table 1.

Summing up 56 higher order moments to the 84 second order moments, each frame is characterized by a set of 140 components in the observation vector.

As the features in the observation vector are mutually correlated at different color channels it is very likely that some components of the observation vector can have a negligible effect regarding texture characterization. Therefore, these components must be located and discarded in order to save computational resources and consequently accelerating the automatic diagnosis process. Under the assumption of Gaussianity, on the parameters that characterize the joint and marginal distributions of the second order textural descriptors, principal components can be obtained by using Principal Component Analysis, which is a well established theory based on linear algebra concepts.

### Implementation details

In order to compute the co-occurrence matrix for the new image, synthesized from the wavelet coefficients from the selected bands, a new algorithm was implemented, to avoid computing co-occurrences in the image corners where no image information exists. The co-occurrence computation was done considering d = 1. A similar algorithm was also developed to calculate the histograms of each frame. A 3.2 GHz Pentium Dual Core processor-based with 1 GB of RAM was used with MATLAB to run the proposed algorithm. The average processing time per frame is about 1 minute, which is unacceptable to real world applications. However, as stated in [37], the reduction of the gradation levels of each color channel from 256 levels to 32 levels does not compromise the texture analysis process. Therefore the processing time per frame drops considerably, to about 1 second per frame, without significant loss of performance. Furthermore it should be noticed that the code was not optimized for speed. However the vast majority of the pixels in the reconstructed image have a level very close to zero, so the most of the information is included in a few, very close, levels, which will lead to a loss of texture information, as very close levels in the 256 levels image are converted to the same level in the 32 levels image. To overcome this limitation, we have to disperse the pixel values to all available range with a simple multiplication by a constant. Therefore the textural information will be present in all the 256 gray levels, and consequently in all the 32 gray levels, after the conversion. The selected color space was the HSV, since it is more similar to the physiological perception of human eye.

#### Classification Scheme

The features were imported into the open source machine learning package WEKA (available at http://www.cs.waikato.ac.nz/ml/weka/). A stratified 10-fold cross-validation procedure was chosen to train a standard multilayer perceptron neural network. The default parameters were kept in the classifier options. The choice of a simple classification scheme, with default parameters, was done in order to make the results more representative of the choice of the features. The 10-fold cross-validation method is a standard procedure to validate machine learning classification outputs and has been found to provide an adequate and accurate estimate of the true error rate [38]. The 10-fold cross-validation algorithm splits the data into 10 partitions, where the proportion of both normal and abnormal frames in each partitions is similar to the entire dataset. The training and classification process is then repeated 10 times, where 9 partitions are used to train and 1 partition is used to assess the classification process. This way, each frame will be used exactly once as test data, allowing to efficiently use the available dataset. In order to have an accurate error estimate, the cross-validation process was repeated 10 times, being this a standard procedure [39].

#### Dataset

**Figure 4:**
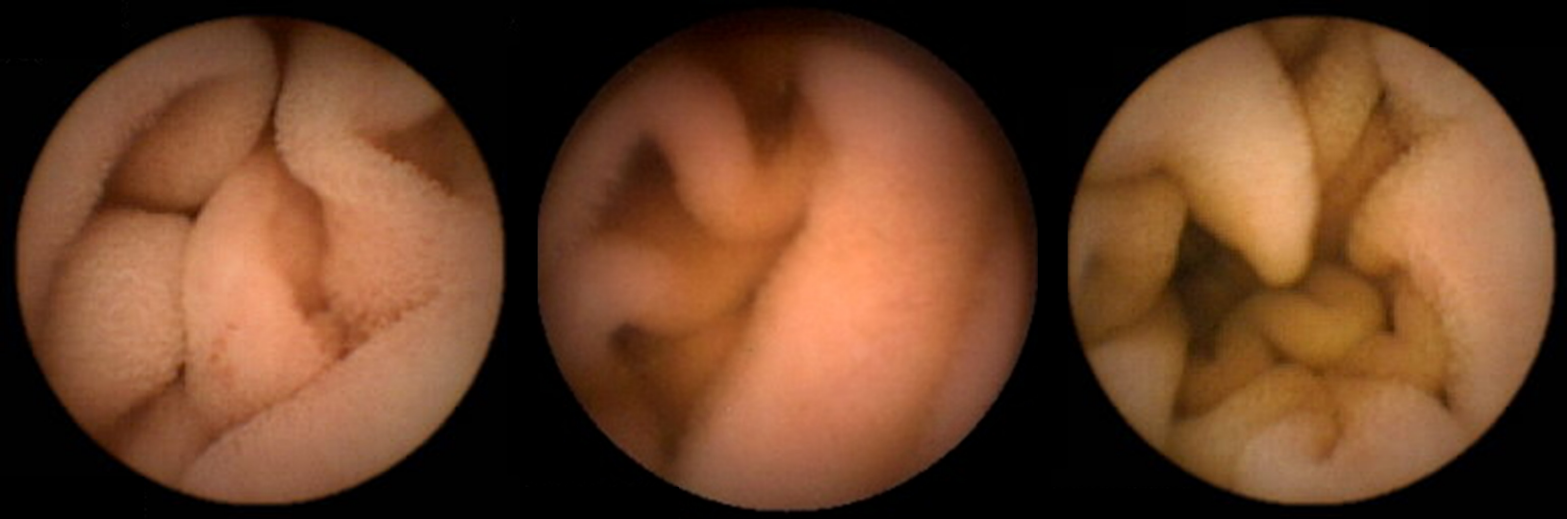
**Examples of normal intestinal tissue frames**. In this figure, several examples of CE frames comprising texture patterns from normal tissues are shown.

**Figure 5:**
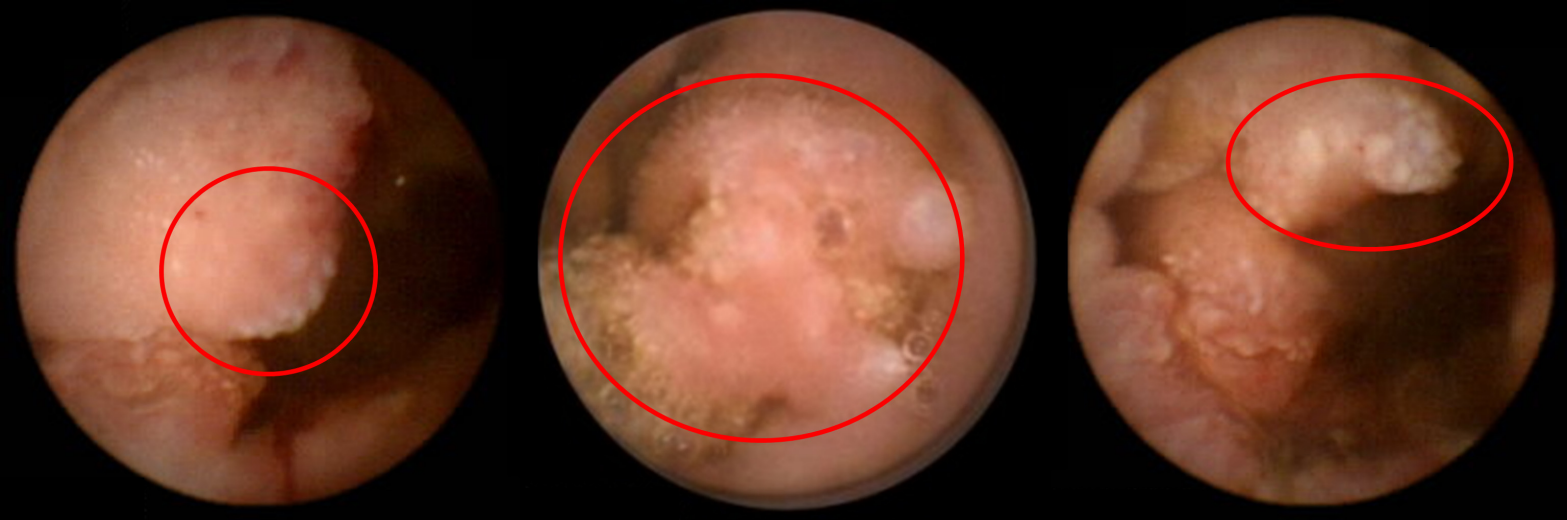
**Examples of abnormal intestinal tissue frames**. In this figure, several examples of CE frames comprising texture patterns from intestinal tumoral tissues are shown.

The experimental dataset contains 700 frames from 14 patients labeled as tumoral frames. These frames were selected by a team of expert physicians from Capucho's Hospital in Lisbon under the criteria of medical unambiguity, which in some cases required invasive complementary examination. Regarding normal frames only 2300 were used in order to balance the amount of data belonging to each class. Roughly twenty percent of these normal frames belong to the 14 patients (33 frames from each one) while roughly 80% of this data belong to 5 normal subjects in order to obtain a large degree of generalization. Figure 4 and Figure 5 show some examples of frames belonging to the dataset.

### Experiments

A baseline system for comparison purposes was firstly implemented to serve as a reference for the current approach. This system is based on the algorithm proposed by Kodogiannis *et al.*, namely the texture descriptors extracted from the histograms/texture spectrums of capsule endoscopic video frames, were extracted for the available dataset [16]. However the classifier did not include the fusion scheme proposed in [16], since the comparison of the methods must be done with the same classification algorithm, in order to evaluate which set of features leads to better classification results. Kodogiannis *et al. *proposed two different schemes to extract features from texture spectra in the chromatic and achromatic domains, namely a structural approach based on the theory of formal languages, which relies on the definition of elementary texture unit TU and texture unit numbers NTU and a statistical approach, where statistical texture descriptors are calculated from the histograms of the RGB and HSV color spaces of capsule endoscopic video frames. A texture unit may be considered as the smallest complete unit which best characterizes the local texture aspect of a given pixel and its neighborhood in all eight directions of a square raster. The texture spectrum histogram is then obtained as the frequency distribution of all the texture units. After the determination of the texture spectrum for each of the RGB and HSV color channels, nine statistical texture descriptors (standard deviation, variance, skew, kurtosis, entropy, energy, inverse difference moment, contrast, and covariance) are extracted from each one, resulting in 54 features. In the statistical approach, the same statistical descriptors are extracted directly from the color histogram of each of the RGB and HSV color channels, with the obvious drawback of not including any kind of information regarding the spatial relationship between the pixels in the image and, therefore, will most probably fail to distinguish between textures with similar distributions of grey levels.

For the approach proposed in the present work, a preliminary version of the proposed algorithm, where no multi-scale information is used, was also implemented, discarding (10) (*i.e. *using just the DWT coefficients of higher frequency). Thus, the added value of the inclusion of the multi-scale information can be properly evaluated.

## Results and Discussion

Table 2 resumes the most relevant results regarding the preliminary version of the proposed algorithm, where no multi-scale information is used. The results are given in statistical terms, and, to test the importance of the higher order statistics, the classification vector for each frame had the second order moments, given by equation (14) or the second and third order moments, given by (14), (15) and (16), just considering the lower scale of the DWT transform. The classification results of the methods proposed in [16] are also shown.

**Table 2:** Classification performance for the baseline methods

	[29]		[16]	
Classification Vector	**2**^**nd **^**order moments**	**2**^**nd **^**and 3**^**rd **^**order moments**	Histogram based features	**N**_**TU **_**based features**
Specificity (*μ *± *σ*%)	88.0 ± 0.5	90.1 ± 0.3	84.7 ± 0.7	88.2 ± 0.3
Sensitivity (*μ *± *σ*%)	88.1 ± 0.4	91.2 ± 0.3	85.5 ± 0.7	89.1 ± 0.5

This table presents the classification performance of the methods used as baseline reference to assess the performance of the proposed algorithm.

Regarding texture characterization it is well known that the most important information lies in the lower to middle scales of the wavelet transform depending on the image intrinsic spatial resolution which is related to the image size. Conventional endoscopy [15] frequently uses higher scales (usually the second) than capsule endoscopy since the images are of different spatial resolution usually at a rate of 16:1. In [27] it was shown that second scale of the WT captures less texture information than the first scale, however results shown that second scale has also an important amount of textural information. It was also shown that processing the original image, which contain all the scales, does not improve the system performance regarding to the case where only the first scale is used. This very important result means that texture information contained in the second scale can not be added in the image domain to the texture information contained in the first scale. However, for a given texture, it is likely that texture coefficients correlate at different scales and can contribute for texture characterization. Therefore the idea that correlating texture descriptors at the 2 lower scales can be an efficient procedure to increase texture information captured by the small scale of the WT, seems to be confirmed by experimental results.

From the analysis of the classification performance of the baseline algorithms, it is clear that regarding to the features effectiveness, the baseline for the current approach is superior to the algorithm proposed in [16], when HOS is included. Therefore, the additional modeling of the non-Gaussianity in the texture descriptors leads to better classification results. One should note that the addition of fourth order moments does not improve significantly the classification performance (data not shown). Note also that to correctly estimate higher order moments, larger amounts of data are needed, and so the classification improvement with the addition of higher order moments will be perhaps more evident in larger datasets.

Modeling joint non-Gaussianity by using third order moments (HOS) improves in this manner the system performance at least for the current database. Additionally the potential for more improvements over larger databases exist since HOS accurate estimates need a large amount of training data. Table 3 shows the results for the current approach of multi-scale texture analysis and the corresponding reduction in the observation vector dimensionality by decorrelating the data using PCA. It is clear comparing tables 2 and 3 that correlations between different scales improve significantly texture information (Sp. = 93.1 ± 0.4%, Se. = 93.9 ± 0.3%) compared to the case where only one scale is used (Sp. = 90.1 ± 0.3%, Se. = 91.2 ± 0.3%).

**Table 3:** Classification performance of the proposed method

	Proposed algorithm		
Classification Vector	Multi-scale analysis	PCA reduction (140 ≫ 70)	PCA reduction (140 ≫ 40)
Specificity (*μ *± *σ*%)	93.1 ± 0.4	92.6 ± 0.2	91.8 ± 0.5
Sensitivity (*μ *± *σ*%)	93.9 ± 0.3	93.3 ± 0.2	92.7 ± 0.2

This table presents the classification performance of the proposed algorithm.

Additionally reducing dimensionality by using PCA reduces performance as expected but only moderately which indicates data correlation in the multi-band features. Even a strong reduction from 140 to 40 coefficients, which is the number of coefficients used in the baseline system for the current approach, maintains a performance slight higher (Sp. = 91.7 ± 0.1%, Se. = 92.9 ± 0.2%) than when the same dimensionality is used without multi-scale analysis (Sp. = 90.1 ± 0.3%, Se. = 91.2 ± 0.3%). This clearly shows that multi-scale analysis can improve texture discrimination regarding classification of tumoral tissue in the small bowel.

An important note should be addressed regarding the specificity of the algorithm. The color of the small bowel tissues may vary between healthy volunteers and patients, which could lead to unbalances in the classification performance of the proposed algorithm in normal tissue in the GI tract of a diseased patient. However, we have not detected a significant difference in the false positive detection rate in our dataset, being this similar in both healthy and diseased subjects. This can be related with the fact that we are not taking first order descriptors associated with the histogram of the image content but rather texture descriptors that are mostly related to the local variation patterns in the image.

Regarding the clinical application of the proposed method, we aim indeed to introduce the developed method in a supervised computer-aided diagnostic system, where the clinicians acts as a final reviewer of the selected frames containing potential textural abnormalities. Therefore, the primary aim of the present work is to alleviate the analysis process of a capsule endoscopy video, reducing the time required by the physician to review the acquired data and to detect abnormalities. This will likely reduce the overall costs associated with this modality. Additionally, it may contribute to a smaller dependency on the physician expertise, allowing users with less experience to achieve better diagnosis performance. Nonetheless, and given the size of CE videos (~50.000 frames), the proposed method will return ~3500 false positives for an entire CE exam. However, the time required to review these selected frames will likely be smaller than the total analysis time required to analyze the entire exam. Nonetheless, it should be stressed that the current performance of the proposed algorithm is still unacceptable for clinical applications in routine practice, since each frame classified as abnormal tissue will imply a deeper analysis by the operating physician, which will take longer than the viewing time in the traditional manual CE exam analysis. This may have a canceling effect on the time savings offered by the proposed algorithm. Therefore, additional efforts aiming the improvement of the classification accuracy should be taken. We are aiming to perform this by including the temporal dynamics to improve the detection rate, by taking into account the classification of neighbor frames to discard wrongly classified frames. In fact, this is a limitation present in most of the CE computer-aided diagnosis algorithms, where the CE frames are analyzed independently. This limitation of the proposed method will be tackled in the near future in order to move towards a software solution capable to be used in clinical routine.

## Conclusions

The results of this paper show that regarding CE video frames classification texture discrimination can be improved by modeling classical texture descriptors in the color-scale plane instead of the color plane as usually assumed by classical approaches. Although a similar approach was proposed in [28] the current statistical model is richer than the model proposed in [28] where HOS was only applied to marginal distributions. However the current approach generates almost twice the dimensionality of the feature vectors that can however be reduced by using PCA with no significant loss in performance, which suggests data redundancy in the joint non-Gaussianity modeling. In spite of this redundancy results presented in this paper are superior to the case where only marginal non-Gaussianity is modeled as was the case in [28]. Hence HOS applied to the joint distribution of classical texture descriptors seems to be effective for texture characterization. Future work will include the augment of the available database, which is important regarding generalization of the presented results, especially when HOS modeling is involved. Different classification schemes will also be subject of future investigation. Another point to be explored in the near future is the temporal dynamics of the texture information, since taking information from neighbor frames may contribute to the improvement of the classification performance.

## Competing interests

The authors declare that they have no competing interests.

## Authors' contributions

DB implemented the algorithm together with DR, being the last in charge also of its test. The experimental data was provided by JR and he provided the medical feedback that drove our work. The work was done under supervision of CL, auxiliated in this task by AT. All authors read and approved the final manuscript.
